# Supplementary figures and images for: Selection and evaluation of reference genes for analysis of mouse (Mus musculus) sex-dimorphic brain development
Source: PeerJ. 2017 Jan 19;5:e2909. doi: 10.7717/peerj.2909 (PMC5251938; doi:10.7717/peerj.2909)

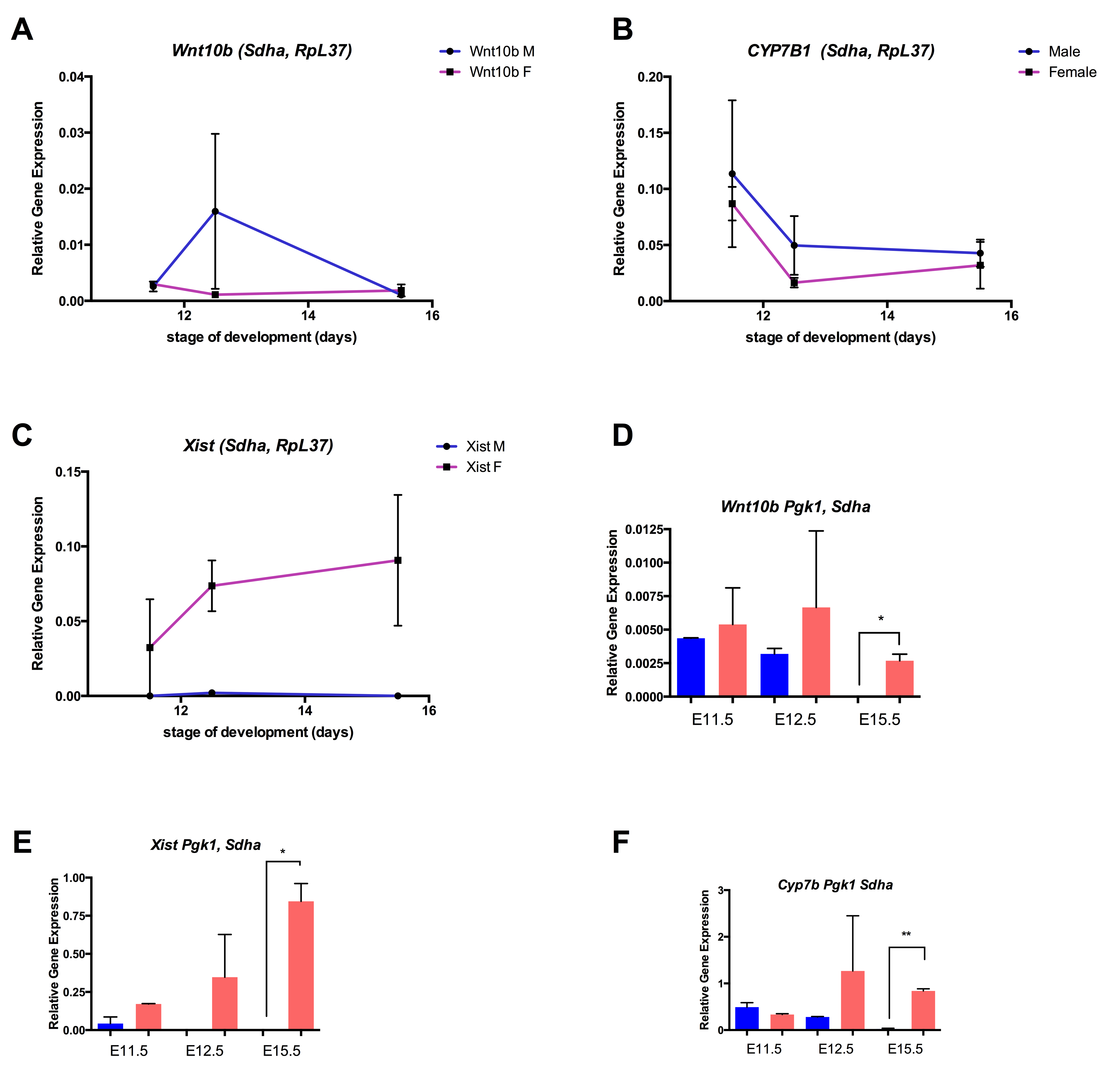

Supplement: Figure S1 — Normalized gene expression for CYP7B1 (A), Wnt10b (B) and Xist (C) using RpL37 and Sdha as combined reference genes. Normalized gene expression for Wnt10b (D), Xist (E) and CYP7B1 using Pgk1 and Sdha as combined reference genes. [file peerj-05-2909-s001.png]
