# Supplementary material for: Selection and evaluation of reference genes for analysis of mouse (Mus musculus) sex-dimorphic brain development
Source: PeerJ. 2017 Jan 19;5:e2909. doi: 10.7717/peerj.2909 (PMC5251938; doi:10.7717/peerj.2909)
Supplement: Table S1 — Name, accession number and oligonucleotide primer sequences used in this study. [file peerj-05-2909-s002.docx]

**Supplementary Table 1:** Name, accession number and oligonucleotide primer sequences used in this study.

| Gene symbol | Accession number | Forward primer sequence (5’-3’) | Reverse primer sequence (5’-3’) |
| --- | --- | --- | --- |
| *Gapdh* | NM_001289726 | TGTGTCCGTCGTGGATCTGA | CCTGCTTCACCACCTTCTTGA |
| *Actb* | NM_007393.5 | GAGGTATCCTGACCCTGAAGTA | CACACGCAGCTCATTGTAGA |
| *Hprt1* | NM_013556.2 | CACAGGACTAGAACACCTGC | GCTGGTGAAAAGGACCTCT |
| *Pgk1* | NM_008828.3 | CTGACTTTGGACAAGCTGGACG | GCAGCCTTGATCCTTTGGTTG |
| *Sdha* | NM_023281.1 | GCTCCTGCCTCTGTGGTTGA | AGCAACACCGATGAGCCTG |
| *Wnt10b* | NM_011718.2 | TCCACTGGTGCTGTTATGTG | GCGAGGCTCACCTTCATTTA |
| *Xist* | NR_001463.3 | GCCCAAAGGGACAAACAATC | GTAGCGAGGACTTGAAGAGAAG |
| *CYP7B1* | NP_004811.1 | CAGTGTCACAGGAAGTGAA | AGAGGAGAACACCCTCCTATT A |
| *Eef2* | NM_007907.2 | CTGGCAGAGGAC ATCGATAAG | GCAACGTCCCACTCATACTT |
| *RpL38* | NM_001048057.1 | TTCGGTCTCATCGCTGTGAGTGT | TCTTGACAGACTTGGCATCCTTCC |
| *Eif3f* | NM_025344.2 | TTATTGTCGGCCAGGAGAAAG | GGGTGAGTCATGCCAACATA |
| *Ppia* | NM_008907.1 | GTGTTCAGGGTGGTGACTTTA | AAGATGCCAGGACCTGTATG |
| *RpL37* | NM_026069.3 | CCAAGCGCAAGAGGAAGTATAA | CATGTCTGAATCTGCGGTAGAC |
| *Sry* | NM_011564.1 | TCTTAAACTCTGAAGAAGAGAC | GTCTTGCCTGTATGTGATGG |
