# Supplementary material for: Selection and evaluation of reference genes for analysis of mouse (Mus musculus) sex-dimorphic brain development
Source: PeerJ. 2017 Jan 19;5:e2909. doi: 10.7717/peerj.2909 (PMC5251938; doi:10.7717/peerj.2909)
Supplement: Table S2 — Amplification efficiency for each oligonucleotide primer set. [file peerj-05-2909-s003.docx]

**Supplementary** **Table 2: Oligonucleotide primer efficiencies.**

| **Gene symbol** | **Efficiency** | **R^2^** |
| --- | --- | --- |
| *Gapdh* | 100 | 0.997 |
| *Actb* | 105 | 0.998 |
| *Hprt1* | 108 | 0.939 |
| *Pgk1* | 105 | 0.988 |
| *Sdha* | 110 | 0.994 |
| *Wnt10b* | 98 | 0.963 |
| *Xist* | 91 | 0.993 |
| *CYP7B1* | 91 | 0.006 |
| *Eef2* | 108 | 0.993 |
| *Eif3f* | 109 | 0.995 |
| *RpL38* | 92 | 0.963 |
| *RpL37* | 102 | 0.976 |
| *Ppia* | 106 | 0.85 |
