# Supplementary material for: Selection and evaluation of reference genes for analysis of mouse (Mus musculus) sex-dimorphic brain development
Source: PeerJ. 2017 Jan 19;5:e2909. doi: 10.7717/peerj.2909 (PMC5251938; doi:10.7717/peerj.2909)
Supplement: Table S3 — Mean Ct values and corresponding standard deviation (SD) of each reference gene with respect to developmental stage/ [file peerj-05-2909-s004.docx]

**Supplementary Table 3.** Mean Ct values with SD of each reference gene and developmental stage

|  | E11.5 | | E12.5 | | E15.5 | All stages |
| --- | --- | --- | --- | --- | --- | --- |
| Male |  |  | |  | |  |
| ***Gapdh*** | 15.77 (1.07) | 16.93 (0.60) | | 19.05 (1.69) | | 17.25 ( 1.66) |
| ***Actb*** | 15.92 (1.38) | 15.46 (0.42) | | 17.69 (1.13) | | 16.36 ( 1.17) |
| ***Hprt1*** | 22.68 (1.51) | 22.81 (1.18) | | 25.57 (1.25) | | 23.69 (1.63) |
| ***Pgk1*** | 20.30 (1.37 | 20.17 (0.18) | | 22.60 (1.22) | | 21.02 (1.36) |
| ***Sdha*** | 21.36 (1.09) | 20.99 (0.66) | | 23.65 (1.17) | | 22.00 (1.44) |
| ***RpL37*** | 16.63 (1.18) | 19.31 (2.30) | | 20.32 (1.50) | | 18.75 (2.24) |
| ***RpL38*** | 18.45 (0.50) | 21.03 (2.33) | | 24.55 (2.85) | | 21.35 (3.23) |
| ***Eef2*** | 19.27 (1.97) | 23.84 (5.70) | | 27.83 (1.26) | | 23.65 (4.82) |
| ***Eif3f*** | 26.51 (2.02) | 28.45 (4.20) | | 32.83 (0.054) | | 30.06 (5.08) |
| ***Ppia*** | 17.88 (1.62) | 20.83 (3.80) | | 24.70 (3.99) | | 21.13 (4.13) |
| Female | | | | | | |
| ***Gapdh*** | 17.00 (4.35 ) | 17.00 ( 2.25) | | 22.08 (5.46) | | 18.69 (2.93) |
| ***Actb*** | 17.39 (3.24) | 15.99 (2.36) | | 20.35 (5.48) | | 17.91 (2.22) |
| ***Hprt1*** | 24.12 ( 3.76) | 23.83 (2.15) | | 27.96 (4.64) | | 25.53 (2.28) |
| ***Pgk1*** | 20.92 (3.85) | 20.59 (0.61) | | 26.10 (5.44) | | 22.53 (3.08) |
| ***RpL37*** | 15.8 (0.49) | 18.78 (2.05) | | 19.39 (3.12) | | 17.99 (2.51) |
| ***RpL38*** | 17.46 (0.32) | 20.61 (2.47) | | 21.67 (3.14) | | 19.92 (2.76) |
| ***Sdha*** | 21.92 (3.67) | 21.953(1.67) | | 26.47 (5.92) | | 23.45 (2.61) |
| ***Eef2*** | 17.67 (1.32) | 24.46 (4.85) | | 24.03 (3.79) | | 22.05 (4.54) |
| ***Eif3f*** | 24.32 (0.85) | 30.59 (5.03) | | 29.96 (3.70) | | 28.30 (4.34) |
| ***Ppia*** | 19.08 (6.41) | 20.81 (4.10) | | 20.85 (4.13) | | 20.25 (4.42) |
