# Supplementary material for: Selection and evaluation of reference genes for analysis of mouse (Mus musculus) sex-dimorphic brain development
Source: PeerJ. 2017 Jan 19;5:e2909. doi: 10.7717/peerj.2909 (PMC5251938; doi:10.7717/peerj.2909)
Supplement: Table S4 — Calculated NormFinder stability values, shaded values are top genes with lowest stability value. [file peerj-05-2909-s005.docx]

**Supplementary Table 4:** NormFinder stability values between male and females at each stage. Shaded values are top genes with lowest stability value.

|  | **E11.5** | **E12.5** | **E15.5** | **Male** | **Female** | **All stages** |
| --- | --- | --- | --- | --- | --- | --- |
| ***ActB*** | 0.309 | 0.748 | 1.585 | 1.131 | 1.09 | 1.573 |
| ***Hprt1*** | 0.683 | 0.724 | 1.308 | 0.942 | 0.929 | 1.436 |
| ***Sdha*** | 0.386 | 0.511 | 1.736 | 1.014 | 1.001 | 1.412 |
| ***Gapdh*** | 0.511 | 0.806 | 1.809 | 0.932 | 1.099 | 1.446 |
| ***Pgk1*** | 0.461 | 0.691 | 1.847 | 1.049 | 1.079 | 1.548 |
| ***Eef2*** | 0.901 | 1.626 | 1.584 | 1.539 | 1.344 | 2.180 |
| ***RpL38*** | 0.830 | 0.253 | 1.835 | 0.862 | 1.0589 | 2.956 |
| ***Eif3f*** | 1.031 | 1.968 | 2.924 | 1.671 | 2.162 | 3.781 |
| ***Ppia*** | 1.156 | 1.011 | 2.121 | 1.289 | 1.525 | 1.856 |
| ***RpL37*** | 0.648 | 0.280 | 1.082 | 0.739 | 1.126 | 1.211 |
| Best two genes  (combined stability value) | *Actb*  and *Sdha*  (0.250) | *Rpl38*  and *Rpl37*  (0.189) | *Pgk1* and  *Eef2*  (0.430) | *Hprt1* and  *Rpl38 (0.559)* | *Rpl38*  and  *Sdha (0.640)* | *Rpl37*  and  *Sdha (0.825)* |
