# Supplementary material for: Selection and evaluation of reference genes for analysis of mouse (Mus musculus) sex-dimorphic brain development
Source: PeerJ. 2017 Jan 19;5:e2909. doi: 10.7717/peerj.2909 (PMC5251938; doi:10.7717/peerj.2909)
Supplement: Table S5 — Shaded values are top two genes with lowest std error values. [file peerj-05-2909-s006.docx]

**Supplementary** **Table 5:** BestKeeper standard error (+/- CP) values between male and females at each stage. Shaded values are top two genes with lowest std error values.

|  | **E11.5** | **E12.5** | **E15.5** | **Male** | **Female** | **All stages** |
| --- | --- | --- | --- | --- | --- | --- |
| ***ActB*** | 1.088 | 1.51 | 1.876 | 1.068 | 1.919 | 1.858 |
| ***Hprt1*** | 1.757 | 1.669 | 1.762 | 1.241 | 1.834 | 1.76 |
| ***Sdha*** | 0.752 | 1.092 | 2.066 | 1.004 | 1.441 | 1.65 |
| ***Gapdh*** | 0.934 | 1.529 | 2.295 | 1.226 | 1.639 | 1.783 |
| ***Pgk1*** | 0.71 | 0.522 | 2.379 | 1.064 | 1.827 | 1.846 |
| ***Eef2*** | 1.868 | 1.373 | 2.454 | 1.655 | 3.391 | 2.974 |
| ***RpL38*** | 0.686 | 0.901 | 2.699 | 1.62 | 1.706 | 2.559 |
| ***Eif3f*** | 2.038 | 4.881 | 5.072 | 3.211 | 4.495 | 4.254 |
| ***Ppia*** | 1.535 | 1.38 | 3.565 | 2.79 | 3.066 | 2.617 |
| ***RpL37*** | 1.032 | 1.248 | 1.52 | 1.237 | 1.702 | 1.514 |
