# Supplementary material for: Selection and evaluation of reference genes for analysis of mouse (Mus musculus) sex-dimorphic brain development
Source: PeerJ. 2017 Jan 19;5:e2909. doi: 10.7717/peerj.2909 (PMC5251938; doi:10.7717/peerj.2909)
Supplement: Table S6 — GeNorm values for each reference gene candidate.Mean M value shaded top two ranked genes. [file peerj-05-2909-s007.docx]

**Supplementary** **Table 6:** GeNorm stability values between sexes at each stage. Mean M value shaded top two ranked genes.

|  | **E11.5** | **E12.5** | **E15.5** | **Male** | **Female** | **All stages** |
| --- | --- | --- | --- | --- | --- | --- |
| ***ActB*** | 0.576 | 0.346 | 0.719 | 0.281 | 0.947 | 0.868 |
| ***Hprt1*** | 0.736 | 0.844 | 3.501 | 0.724 | 0.856 | 1.638 |
| ***Sdha*** | 0.248 | 0.552 | 1.141 | 0.323 | 0.591 | 0.868 |
| ***Gapdh*** | 0.448 | 0.346 | 4.592 | 0.559 | 0.591 | 3.546 |
| ***Pgk1*** | 0.248 | 1.045 | 0.719 | 0.281 | 1.062 | 1.067 |
| ***Eef2*** | 2.057 | 2.389 | 2.762 | 2.342 | 2.876 | 3.101 |
| ***RpL38*** | 1.807 | 1.47 | 2.935 | 1.617 | 1.85 | 2.571 |
| ***Eif3f*** | 2.223 | 2.932 | 6.075 | 2.707 | 3.38 | 4.401 |
| ***Ppia*** | 2.44 | 1.992 | 3.084 | 2.01 | 2.547 | 2.845 |
| ***RpL37*** | 1.46 | 1.659 | 2.239 | 1.195 | 2.212 | 2.29 |
