# Supplementary material for: Selection and evaluation of reference genes for analysis of mouse (Mus musculus) sex-dimorphic brain development
Source: PeerJ. 2017 Jan 19;5:e2909. doi: 10.7717/peerj.2909 (PMC5251938; doi:10.7717/peerj.2909)
Supplement: Table S7 — Average standard deviation (of delta Ct values) for reference genes, sex and developmental stages. [file peerj-05-2909-s008.docx]

**Supplementary Table 7:** Average standard deviation (of delta Ct values) for reference genes, sex and developmental stages.

|  | ***E11.5*** | ***E12.5*** | ***E15.5*** | ***Male*** | ***Female*** | ***All stages*** |
| --- | --- | --- | --- | --- | --- | --- |
| *Gapdh* | 2.09 | 2.5 | 8.16 | 2.29 | 2.82 | 5.13 |
| *Actb* | 1.98 | 1.98 | 4.7 | 2.29 | 2.87 | 3.57 |
| *Hprt1* | 2.31 | 2.6 | 5.25 | 2.3 | 2.78 | 3.72 |
| *Pgk1* | 1.98 | 1.92 | 5.01 | 2.19 | 2.91 | 3.73 |
| *Sdha* | 1.87 | 2.25 | 4.73 | 2.15 | 2.67 | 3.39 |
| *Eef2* | 2.76 | 4.02 | 5.17 | 3.51 | 4.14 | 4.71 |
| *RpL38* | 2.65 | 2.36 | 5.21 | 2.63 | 3.12 | 3.88 |
| *Eif3f* | 3.01 | 5.11 | 12.01 | 4.17 | 5.44 | 7.82 |
| *Ppia* | 3.31 | 3.08 | 5.54 | 3.14 | 3.92 | 4.3 |
| *RpL37* | 2.5 | 2.45 | 4.98 | 2.41 | 3.22 | 3.77 |
