# Supplementary material for: Selection and evaluation of reference genes for analysis of mouse (Mus musculus) sex-dimorphic brain development
Source: PeerJ. 2017 Jan 19;5:e2909. doi: 10.7717/peerj.2909 (PMC5251938; doi:10.7717/peerj.2909)
Supplement: Table S8 — Summary of rankings for comparing gene expression between male and female samples at each embryonic stage. [file peerj-05-2909-s009.docx]

**Supplementary** **Table 8**: Summary of rankings across developmental stages by sex.

| Ranking | Male |  |  | Female |  |  |
| --- | --- | --- | --- | --- | --- | --- |
|  | BestKeeper | NormFinder | GeNorm | BestKeeper | NormFinder | GeNorm |
| 1 | *Sdha* | *Rpl37* | *Actb/Pgk1* | *Sdha* | *Hprt1* | *Sdha/Gapdh* |
| 2 | *Actb* | *Rpl38* | *-* | *Gapdh* | *Sdha* | *-* |
| 3 | *Gapdh* | *Gapdh* | *Sdha* | *Rpl37* | *Rpl38* | *Hprt1* |
| 4 | *Rpl37* | *Hprt1* | *Gapdh* | *Rpl38* | *Actb* | *Actb* |
| 5 | *Hprt1* | *Sdha* | *Hprt1* | *Pgk1* | *Gapdh* | *Pgk1* |
| 6 | *Pgk1* | *Pgk1* | *Rpl37* | *Hprt1* | *Pgk1* | *RpL38* |
| 7 | *Eef2* | *Actb* | *RpL38* | *Actb* | *Rpl38* | *RpL37* |
| 8 | *Rpl38* | *Ppia* | *Ppia* | *Ppia* | *Eef2* | *Ppia* |
| 9 | *Ppia* | *Eef2* | *Eef2* | *Eef2* | *Ppia* | *Eef2* |
| 10 | *Eif3f* | *Eif3f* | *Eif3f* | *Eif3f* | *Eif3f* | *Eif3f* |
